# Supplementary material for: Role and mechanism of NCAPD3 in promoting malignant behaviors in gastric cancer
Source: Front Pharmacol. 2024 Apr 22;15:1341039. doi: 10.3389/fphar.2024.1341039 (PMC11070777; doi:10.3389/fphar.2024.1341039)
Supplement: Supplementary file 11 [file DataSheet2.ZIP › GSEA/immunologic/my_analysis.Gsea.1599462003817/GSE29618_BCELL_VS_PDC_UP.html]

Details for gene set GSE29618\_BCELL\_VS\_PDC\_UP[GSEA]

|  || Dataset | filtered\_dataset.sample\_info.cls#WT\_versus\_NCAPD3\_MUT |
| Phenotype | sample\_info.cls#WT\_versus\_NCAPD3\_MUT |
| Upregulated in class | NCAPD3\_MUT |
| GeneSet | GSE29618\_BCELL\_VS\_PDC\_UP |
| Enrichment Score (ES) | -0.51555306 |
| Normalized Enrichment Score (NES) | -2.6058013 |
| Nominal p-value | 0.0 |
| FDR q-value | 0.0056079156 |
| FWER p-Value | 0.032 |
Table: GSEA Results Summary

  

Fig 1: Enrichment plot: GSE29618\_BCELL\_VS\_PDC\_UP      
 Profile of the Running ES Score & Positions of GeneSet Members on the Rank Ordered List

  

| SYMBOL | TITLE | RANK IN GENE LIST | RANK METRIC SCORE | RUNNING ES | CORE ENRICHMENT || 1 | 6672 | SP100 | 185 | 0.689 | -0.0831 | No |
| 2 | 84159 | ARID5B | 699 | 0.379 | -0.4259 | No |
| 3 | 25825 | BACE2 | 756 | 0.344 | -0.4411 | No |
| 4 | 2872 | MKNK2 | 860 | -0.269 | -0.4958 | Yes |
| 5 | 6515 | SLC2A3 | 873 | -0.283 | -0.4837 | Yes |
| 6 | 51278 | IER5 | 874 | -0.283 | -0.4630 | Yes |
| 7 | 571 | BACH1 | 884 | -0.300 | -0.4474 | Yes |
| 8 | 694 | BTG1 | 901 | -0.326 | -0.4350 | Yes |
| 9 | 5163 | PDK1 | 982 | -0.394 | -0.4639 | Yes |
| 10 | 23499 | MACF1 | 989 | -0.398 | -0.4390 | Yes |
| 11 | 7277 | TUBA4A | 997 | -0.404 | -0.4144 | Yes |
| 12 | 10365 | KLF2 | 1000 | -0.405 | -0.3860 | Yes |
| 13 | 55589 | BMP2K | 1017 | -0.417 | -0.3670 | Yes |
| 14 | 9308 | CD83 | 1025 | -0.423 | -0.3410 | Yes |
| 15 | 8609 | KLF7 | 1071 | -0.457 | -0.3399 | Yes |
| 16 | 1604 | CD55 | 1090 | -0.468 | -0.3186 | Yes |
| 17 | 1889 | ECE1 | 1091 | -0.468 | -0.2842 | Yes |
| 18 | 3669 | ISG20 | 1097 | -0.475 | -0.2529 | Yes |
| 19 | 7204 | TRIO | 1120 | -0.494 | -0.2325 | Yes |
| 20 | 3134 | HLA-F | 1139 | -0.506 | -0.2083 | Yes |
| 21 | 330 | BIRC3 | 1224 | -0.600 | -0.2250 | Yes |
| 22 | 23508 | TTC9 | 1271 | -0.660 | -0.2098 | Yes |
| 23 | 80853 | KDM7A | 1311 | -0.714 | -0.1855 | Yes |
| 24 | 57157 | PHTF2 | 1334 | -0.755 | -0.1459 | Yes |
| 25 | 1649 | DDIT3 | 1341 | -0.779 | -0.0930 | Yes |
| 26 | 113791 | PIK3IP1 | 1345 | -0.787 | -0.0373 | Yes |
| 27 | 7462 | LAT2 | 1399 | -1.138 | 0.0079 | Yes |
Table: GSEA details [plain text format]

  

Fig 2: GSE29618\_BCELL\_VS\_PDC\_UP      
 Blue-Pink O' Gram in the Space of the Analyzed GeneSet

  

Fig 3: GSE29618\_BCELL\_VS\_PDC\_UP: Random ES distribution      
 Gene set null distribution of ES for **GSE29618\_BCELL\_VS\_PDC\_UP**
